# Artificial Intelligence‐Empowered Single‐Cell Phenotyping for Rapid, Automated Pathogen Diagnostics

**DOI:** 10.1002/advs.77334

**Published:** 2026-08-21

**Authors:** Sabita Khadka, Bushra Raahat, Spyros Tragoudas, Hui Li

**Affiliations:** ^1^ School of Electrical Computer, and Biomedical Engineering Southern Illinois University Carbondale Illinois USA

**Keywords:** deep learning analysis, infection diagnosis, rapid antimicrobial susceptibility test, single‐cell detection

## Abstract

Accurate bacterial identification and antimicrobial susceptibility testing (AST) are essential for timely clinical decision‐making. Conventional diagnostic methods typically require 24–48 h, leading to inappropriate empirical therapy, accelerating antimicrobial resistance, and worsening patient outcomes. Here, we present an integrated diagnostic platform that combines microfluidic single‐cell bacterial detection with artificial intelligence (AI)‐driven analysis for rapid phenotypic AST. The microfluidic device enables stable time‐lapse detection of individual bacterial cells under designated, controlled antibiotic exposure, with a U‐Net–based AI model that automatically segments and quantifies the drug response of individual bacterial cells using phase‐contrast microscopy images. The trained model identified 96% of individual *Escherichia coli* (*E. coli*) cells and showed no false‐positive predictions on bacteria‐free images. Applied to AST, the integrated system quantified dose‐dependent growth inhibition of *E. coli* exposed to ciprofloxacin and trimethoprim/sulfamethoxazole, yielding resistance profiles, along with minimum inhibitory concentrations (MICs), within 2 h. These results are consistent with standard broth microdilution, demonstrating the potential of integrating microfluidics and AI for rapid, automated, and single‐cell–resolved pathogen diagnosis. Beyond rapid AST, the integrated microfluidic‐AI platform was further evaluated for automated identification of targeted infections, including *E. coli* and *Staphylococcus aureus* (*S. aureus*) in the presence of blood matrices.

## Graphical abstract

This work presents an integrated diagnostic platform that combines microfluidic single‐cell bacterial detection with artificial intelligence‐driven analysis for rapid antimicrobial susceptibility testing. Single‐cell phenotyping enables assessment of antibiotic response in only a few cell replication cycles, while AI analysis supports precise bacterial detection, quantification, and time‐resolved growth monitoring in an automated manner.

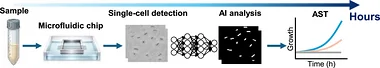

## 1 Introduction

Bacterial infections remain a major global healthcare challenge and one of the leading causes of morbidity and mortality. They account for more than 2.8 million infections each year in the United States alone, leading to 35 000+ deaths and over $20 billion in healthcare costs [1, 2]. This crisis is even more severe globally [2]. The main reason driving this global healthcare crisis is the emergence and spread of antimicrobial‐resistant (AMR) bacteria that are difficult to treat with commonly used antibiotics [3].

Often, antibiotic misuse results from the lack of prompt delivery of precise diagnostic results and antibiotic prescriptions [4]. Specifically, current standard pathogen diagnostics require time‐consuming phenotypic culture‐based procedures, such as Chromogenic culture for pathogen identification and overnight culture of micro‐dilutions for antimicrobial susceptibility testing (AST), which typically take several days (Figure 1a) [5]. During this lengthy culture period, physicians often rely on empirical broad‐spectrum antibiotic therapy based on the worst‐case scenarios, which can lead to unnecessary and inappropriate treatment. Another widely used methodology is nucleic acid detection – namely, genotypic diagnosis, which can amplify and identify designated nucleic acids in vitro within hours by exempting the slow biological replication in nature, such as polymerase chain reaction (PCR), thereby enabling rapid pathogen identification and prediction of AST via its associated gene markers [6, 7, 8, 9, 10]. Nevertheless, the presence of such gene markers neither necessarily translates to phenotypic resistance nor allows the determination of bacterial viability (e.g., live/dead), compromising the delivery of precise treatment suggestions [11]. These practices compromise clinical outcomes, escalate mutation pressure for antibiotic resistance, and facilitate the transmission and dissemination of resistant bacteria, thereby systematically increasing hospitalizations, morbidity, and associated costs related to AMR.

**FIGURE 1:**
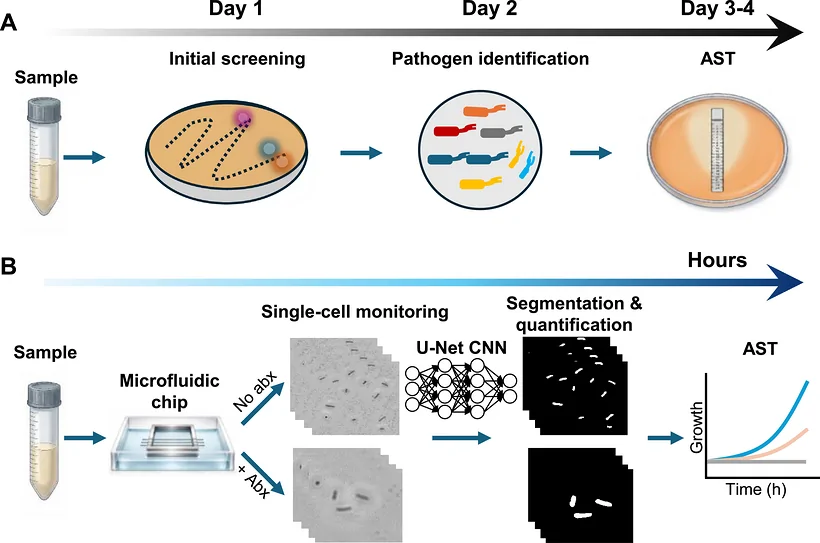
Overview of the integrated microfluidic and AI‐based diagnostic platform for rapid bacterial detection and antimicrobial susceptibility testing. (A) Conventional antimicrobial susceptibility testing (AST) workflow. Bacteria are cultured overnight, standardized, exposed to antibiotics, and evaluated for growth inhibition to determine the minimum inhibitory concentration (MIC). (B) AI‐empowered single‐cell phenotyping for rapid, automated pathogen diagnosis. Bacteria are loaded into a microfluidic chip that enables confinement and monitoring of individual cells. Time‐lapse phase‐contrast images are acquired and analyzed using a U‐Net–based deep learning model for automated bacterial detection and quantification at the single‐cell level. The resulting cell counts are used to generate growth curves under control and antibiotic‐treated conditions for AST.

Microfluidic systems have attracted significant attention in developing rapid pathogen diagnostics [4, 12, 13, 14]. In particular, they excel in discretizing bacteria in confined tiny volumes, such as microwells, microchannels, and droplets, shrinking bulky benchtop experiments into an array of single‐cell detection assays [15, 16, 17, 18, 19, 20]. Consequently, the localized bacterial concentration in the target‐containing assays is substantially elevated, enabling detection with unprecedented quantitative precision and resolution compared to the bulk assays [21, 22, 23]. Such assays thus perform subsequent pathogen identification with single‐cell/molecular resolution [24, 25, 26, 27] and phenotypical AST by interrogating those individual bacteria that are locally well‐concentrated and targeted [28, 29, 30, 31], typically with a prompt turnaround. For instance, AST has been demonstrated within a few hours by detecting single‐cell bacterial growth in the presence/absence of antibiotics, which dramatically shortens the traditional diagnosis that requires days [29, 30, 31, 32, 33, 34, 35, 36]. Although rapid and effective, the quantitative tracking of single‐cell growth in microscopy images is typically conducted manually—for example, using ImageJ—which is, however, labor‐intensive, slow, and may be prone to errors. Traditional thresholding can be semiautomated, but its performance depends on the intensity separation between bacteria and the background and can degrade in phase‐contrast images with uneven illumination, optical halos, and other noise. In addition, these limitations become more pronounced in clinically relevant matrices, where small bacterial cells must be distinguished from heterogeneous host cells, debris, out‐of‐focus particles, and variable backgrounds—all of which introduce substantial noise [37, 38, 39, 40]. Therefore, it highlights an unmet need for an integrated, scalable system for single‐cell bacterial pathogen diagnosis in routine use.

Deep‐learning architectures have substantially advanced the automated analysis of bacterial and cellular images. DeepLabv3+ performs semantic segmentation by combining multiscale contextual feature extraction with boundary refinement [41]. Mask R‐CNN performs instance segmentation by detecting individual objects and predicting object‐specific masks [42]. However, these architectures are not necessarily designed for label‐free detection and quantitative counting of small bacterial objects in noisy phase‐contrast images. In contrast, U‐Net is well‐suited for this task because its encoder–decoder architecture generates pixel‐level masks, while skip connections preserve the fine spatial details needed for target localization [43, 44]. As a result, U‐Net and U‐Net‐like architectures have been widely adopted in bacterial and cellular image‐analysis workflows, including DeLTA, MiSiC, Cellpose/Omnipose, and StarDist [39, 45, 46, 47, 48]. These tools have been utilized in specific contexts, such as mother‐machine microfluidics, agarose‐pad imaging, dense bacterial communities, and antibiotic‐induced morphological analysis. However, to our knowledge, these existing approaches have not been specifically validated for label‐free single‐cell bacterial detection, quantification, and phenotyping in diagnostic workflows involving clinically relevant biological matrices.

In this work, we developed a microfluidic platform combined with automated image analysis using a deep‐learning U‐Net convolutional neural network [43, 44] to perform pathogen diagnosis at the single‐cell level (Figure 1b). The microfluidic device is capable of monitoring the morphological changes of *Escherichia coli* (*E. coli*) at the single‐cell level under exposure to a panel of antibiotic concentrations. The U‐Net model accurately segments and quantifies individual bacteria, allowing dynamic measurement of bacterial growth and their morphological changes. This platform was utilized to perform AST for two commonly used antibiotics—ciprofloxacin and trimethoprim/sulfamethoxazole, with resistance profiles and MICs determined within 2 h, which fully match the standard broth microdilution methods. Moreover, this microfluidic‐AI platform was further evaluated for targeted bacterial identification under complex biological conditions—including the identification of individual *E. coli* and *S. aureus* cells in the presence of blood cells—further demonstrating its feasibility for future use. Compared to standard pathogen diagnosis, this microfluidic‐AI diagnostic platform represents a fast, reliable alternative, enabling automated analysis, reducing turnaround time, and minimizing manual error.

## 2 Results

### 2.1 Microfluidic Platform for Single‐Cell AST

A rapid diagnostic platform that integrates microfluidics and artificial intelligence for bacterial detection and antimicrobial susceptibility testing, including minimum inhibitory concentration (MIC) determination (Figure 2a), was manufactured. The microfluidic device was assembled from a glass substrate, double‐sided adhesive tape, a coverslip, and laser‐cut acrylic support layers (Figure 2b). All patterned layers were fabricated using a laser cutter (Epilog Laser Systems). Each microfluidic device contains three parallel channels with dimensions of 200 µm × 5 µm × 20 mm (W × H × L). These dimensions were designed for effective single‐cell bacterial trapping, imaging, and analysis. In particular, the 5 µm channel height restricts bacterial displacement along the *Z*‐axis and confines most cells within a narrow focal range, thereby facilitating continuous single‐cell monitoring under a microscope setting (Figure S1). No obvious channel collapse or tape‐induced deformation was observed under the experimental conditions used in this study.

**FIGURE 2:**
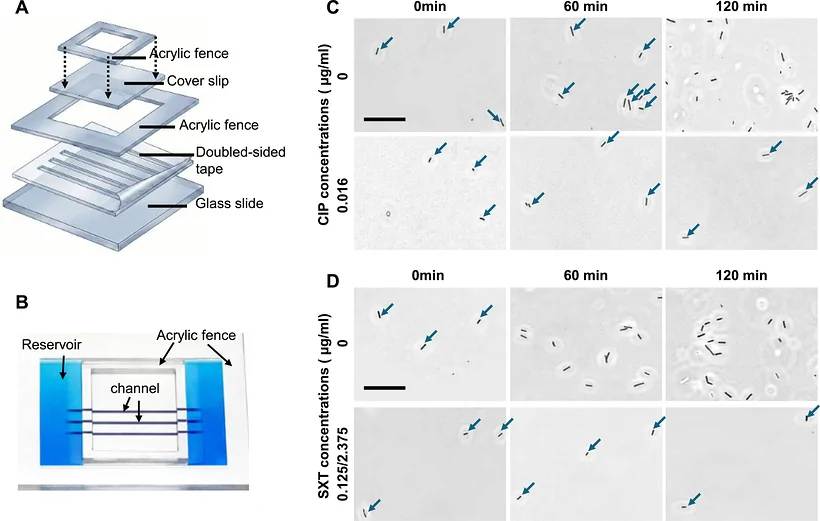
Microfluidic device for time‐resolved single‐cell bacterial phenotyping. (A) Exploded schematic of the multilayer device architecture, showing the glass substrate, laser‐patterned adhesive‐tape microchannels, coverslip, and acrylic support layers. (B) Assembled microfluidic chip with inlet/outlet reservoirs and microchannels for bacterial trapping and observation. Blue dye highlights the fluidic path and channel geometry. (C) Time‐lapse images showing *E. coli* cell division in the absence of antibiotics (top) and complete growth inhibition in the presence of ciprofloxacin at 0.016 µg/mL (bottom), demonstrating stable and continuous single‐cell monitoring for AST. (D) Time‐lapse images *E. coli* cell division in the absence of antibiotics (top) and complete growth inhibition in the presence of sulfamethoxazole/trimethoprim at 0.125/2.375 µg/mL (bottom). Scale bar: 10 µm.

Single‐cell AST was performed using a model *E. coli* strain (ATCC 25922) and two commonly used antibiotics—ciprofloxacin (CIP) and trimethoprim/sulfamethoxazole (SXT). Log‐phase *E. coli* at 10^7^ CFU/mL, with defined antibiotic concentrations, was introduced into the channels by capillary loading. At this bacterial concentration, there were approximately 5–20 spatially separated bacterial cells within each microscopy field of view (i.e., 180 × 120 µm), providing a sufficient proportion of bacteria‐containing images for reproducible single‐cell tracking and quantification within a practical acquisition period. Upon bacterial loading, medium was added to both ends of each channel to maintain hydrodynamic balance; applying equal hydrostatic pressure at the inlet and outlet minimized net flow, reduced cell displacement, and prevented bacterial washout. Under this static‐flow condition, no substantial translational movement that interfered with bacterial detection or quantification was observed during the experiments. Thus, most of the bacterial cells remained near the same location throughout time‐lapse acquisition, so changes in cell number primarily reflected cell division rather than drift (Figures S2 and S3). This stable imaging environment enabled reliable population tracking and supported accurate single‐cell bacterial quantification over time.

Upon sample loading, time‐lapse images were acquired, and bacterial growth in the presence of CIP was quantified through single‐cell bacterial counting (Figure 2c and Figure S2). In antibiotic‐free controls, bacterial cell numbers increased exponentially, consistent with exponential growth. With increasing CIP concentrations, bacterial growth and inhibition were dose‐dependent: bacteria exhibited slower cell division, filamentation without division, and complete arrest of both filamentation and cell division, which aligns with CIP's bactericidal mechanism in disrupting DNA replication and cell division [49]. For MIC determination, cell division was quantified regardless of filamentation, and the MIC was defined as the lowest antibiotic concentration at which cell division was completely suppressed. The data indicate that the tested *E. coli* strain was susceptible to CIP, with a MIC of 0.016 µg/mL. Similarly, SXT likewise produced progressive inhibition as concentration increased (Figure 2d and Figure S3). At subinhibitory concentrations, growth slowed but continued; at 0.125/2.375 µg/mL (trimethoprim/sulfamethoxazole ratio), cell division was no longer observed, defining the MIC. For both antibiotics, growth‐based MIC values obtained from triplicate single‐cell detection matched broth microdilution results, with no detectable adverse inoculum effect observed under the experimental conditions of this study [50] (Figure S4). Importantly, the microfluidic assay generated phenotypic readouts within 2 h, dramatically shortening the 24–48 h timeframe required for traditional AST.

### 2.2 U‐Net Model for Single Bacterial Cell Detection, Counting, and AST Automation

To automate bacterial detection and quantification, a U‐Net–based convolutional neural network for the semantic segmentation of phase‐contrast microscopy images (Figure 3a) was implemented. The training dataset included images containing approximately 1,200 *E. coli* cells, as well as negative‐control images without *E. coli* and images containing *Staphylococcus aureus*. The model achieved 99.81% training accuracy and 99.78% validation accuracy (Figure 3b,c), indicating highly consistent performance on both seen and unseen data. The corresponding training and validation losses were 0.0056 and 0.0067, respectively, suggesting effective learning without evidence of overfitting. Segmentation quality was further supported by a Dice coefficient of 0.8437 and a Jaccard index of 0.7297, confirming strong spatial agreement between predicted masks and manual annotations. Recall and precision values of 0.836 and 0.8516, respectively, indicate that the model detected true bacterial regions with high sensitivity while maintaining a low false‐positive rate.

**FIGURE 3:**
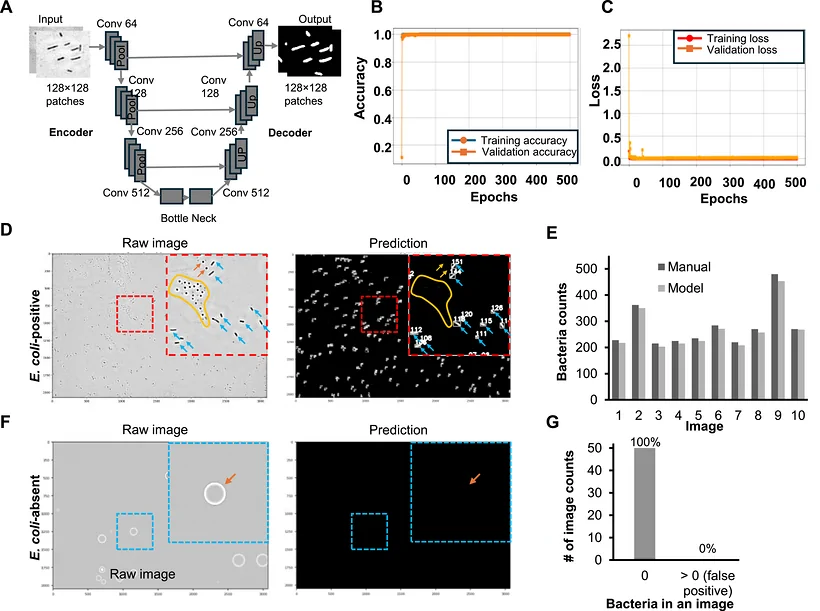
U‐Net model architecture, training performance, and bacterial detection accuracy. (A) U‐Net convolutional neural network used for automated semantic segmentation of bacterial cells in phase‐contrast images. (B) Training and validation accuracy over 500 epochs. (C) Training and validation loss over 500 epochs. (D) Representative phase‐contrast image and corresponding U‐Net prediction output showing accurate bacterial detection. Red rectangles indicate the magnified regions; blue arrows indicate *E. coli* cells, and orange highlights indicate background noise or imaging artifacts. (E) Comparison of bacterial counts obtained by manual counting and U‐Net–based automated analysis across 10 independent test images. (F) Negative‐control image without *E. coli* cells and the corresponding U‐Net prediction mask. The blue rectangle indicates a magnified region containing a noise spot, which was correctly excluded as a non‐bacterial object. (G) Histogram showing the distribution of predicted bacterial counts across 50 bacteria‐absent images.

The U‐Net model was next evaluated for its detection sensitivity and specificity of identifying bacteria in an independent image set. Ten images containing visible *E. coli*, with ground‐truth counts ranging from 215 to 480 cells per image, were analyzed to assess detection accuracy. The model generated predicted masks that separated bacterial regions from background and enabled automated cell counting (Figure 3d). Across these test images, the model correctly identified 96% of individual bacteria, with a standard deviation of ±1% (Figure 3d,e). To assess specificity, the model was also tested on 50 images confirmed to be free of *E. coli*. In this negative‐control dataset, the model predicted zero bacterial cells in all images, with no false‐positive detections, demonstrating its ability to distinguish true bacterial signals from background noise and imaging artifacts (Figure 3f,g). This level of accuracy is critical for downstream antimicrobial susceptibility testing, where reliable quantification of growth inhibition is required for MIC determination.

The trained U‐Net model was employed to re‐analyze the single‐cell AST data in an automated fashion. By segmenting and counting individual *E. coli* cells in time‐lapse images, the model enabled precise, real‐time tracking of bacterial proliferation at the single‐cell level. Across all experimental conditions, the model's predictions revealed a clear dose‐dependent response to CIP exposure, with growth progressively inhibited at higher antibiotic concentrations (Figure 4a and Figure S5). The growth profiles were quantified by normalizing the bacterial cell numbers over time, which is the cell number measured at each time point divided by the initial cell number at (t = 0). The results suggest that the U‐Net—based cell number predictions closely match ground‐truth numbers obtained via manual image inspection and cell counting using ImageJ, with minimal discrepancies, demonstrating its robustness and reliability for high‐resolution image analysis (Figure 4 vs Figure 2). Based on the growth profiles generated by the model, the MIC of CIP against the tested *E. coli* strain was determined to be 0.016 µg/mL, consistent with that obtained by manual analysis (Figure 4b). Moreover, this U‐Net model was employed to analyze single‐cell AST results for SXT, with *E. coli* reproduction quantitatively measured by normalized bacterial cell numbers across a series of SXT concentrations (Figure 4c and Figure S6). Similarly, the results indicate strong agreement between the U‐Net model and ground‐truth results obtained through ImageJ‐based manual analysis across all antibiotic conditions, yielding the MIC of SXT at 0.125/2.375 µg/mL (Figure 4d). These results collectively suggest that our U‐Net model is capable of quantitatively analyzing and interpreting single‐cell AST results in an accurate, automated fashion.

**FIGURE 4:**
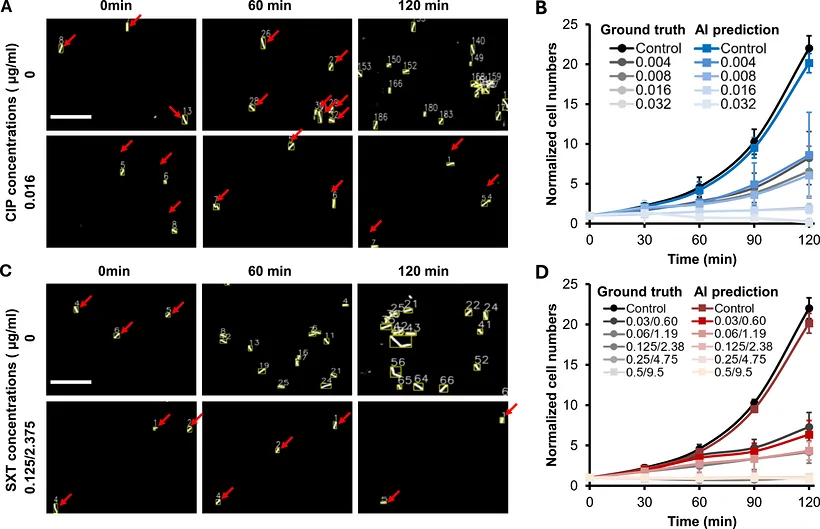
U‐Net‐enabled automated bacterial detection and growth analysis for single‐cell AST. (A) U‐Net segmentation of time‐lapse images of *E. coli* growth in the absence of antibiotics (top) and under CIP treatment at the minimum inhibitory concentration (MIC; bottom), corresponding to the manually analyzed images shown in Figure 2C. (B) Automated single‐cell quantification of *E. coli* cell numbers across a panel of CIP concentrations using U‐Net segmentation, benchmarked against ground‐truth manual image inspection and cell counting using ImageJ. (C) U‐Net segmentation of representative time‐lapse images from the SXT assay, showing untreated bacterial growth (top) and growth inhibition at the MIC condition (bottom), corresponding to the manually analyzed images shown in Figure 2D. (D) U‐Net‐derived growth profiles across the SXT concentration panel via the normalized cell numbers, demonstrating automated quantification of antibiotic‐dependent inhibition and agreement with manual analysis using ImageJ. Scale bar: 10 µm. Data represent mean ± SEM; *n* = 3.

### 2.3 Bacterial Detection in the Presence of Blood Matrices

The U‐Net model was evaluated for bacterial identification and quantification under complex biological conditions, particularly the bacteremia model, in which bacteria are present in the bloodstream, triggering dysregulated host immune response and resulting in severe, acute organ failure and high mortality [51, 52, 53]. More specifically, the U‐Net model was developed to identify *E. coli* and *Staphylococcus aureus* (*S. aureus*), the two most prevalent bacteremia‐causing species, in the presence of blood cells. For this experiment, samples were prepared with bacteria and blood cells at approximately 10^7^–10^8^ cells/mL to evaluate bacterial recognition in the presence of blood‐derived background components. These relatively high bacterial concentrations were selected for proof‐of‐concept image analysis and were not intended to reproduce the low bacterial burdens observed in clinical bacteremia. The trained model was directly employed for *E. coli* detection; whereas it was enhanced with a morphology‐adaptive post‐analysis to detect *S. aureus*, which often appear conjugated in pairs and clusters. Briefly, the U‐Net‐generated binary masks were post‐processed using watershed segmentation based on distance transforms to decouple the individual objects. The projected area of each object within the 95% confidence interval of the benchmark area (i.e., mean ± 2 S.D.) of an individual *S. aureus* was considered a single bacterium; while larger ones were considered clusters and converted into estimated bacterial counts using area‐based proportional assignment. This enhanced method enabled consistent quantification of coccoid bacteria without retraining the U‐Net model, providing a practical means to adapt the platform to different bacterial shapes. Of note, although the post‐analysis leverages morphology to enhance detection, this step should be distinguished from the feature‐learning process of the convolutional network. Instead, the network learns latent representations directly from the training images, which likely encode gross cellular morphology together with additional image characteristics.

The trained U‐Net model was used to identify and quantify targets of interest—*E. coli* and *S. aureus*—across 5 biological samples, including samples containing *E. coli* only, *S. aureus* only, blood cells, blood cells + *E. coli*, and blood cells + *S. aureus*, representing distinct scenarios to evaluate the detection sensitivity and specificity of the U‐Net (Figure 5a and Figure S7). For each biological sample, 10 images were analyzed, with U‐Net–based identification and quantification benchmarked against ground‐truth values established by manual counting, with the prediction‐to‐reference ratio defined as the model‐predicted cell number divided by the corresponding ground‐truth manual count (Figure S8). When applied to test samples containing bacteria, the U‐Net model detected all *E. coli* and *S. aureus* in culture‐only samples, but overestimated *E. coli* by 3% (Figure 5a, left two, and Figure 5b; and Figure S8a,b). These results suggest its high detection sensitivity. No false‐positive detections were observed in samples containing only blood cells, indicating the model's high specificity in distinguishing targeted bacteria from other biological matrices such as blood cells (Figure 5a, middle, and Figure 5b; and Figure S8c). Notably, bacterial detection sensitivity and specificity also remained high in samples co‐existing with bacterial targets and blood cells. Specifically, 100% *E. coli* and 91% *S. aureus* were correctly identified in the presence of blood cells at a comparable concentration to the bacterial cells, yet no blood cell was misclassified as bacteria (Figure 5a, right two, Figure 5b; and Figure S8d,e). The slight underestimation for *S. aureus* likely reflects small differences in how clustered cells were separated by manual vs. automated analysis. In the presence of high cluster density and overlap, the model may also underperform. Such accurate, and importantly, automated detection of targeted bacteria in the blood matrix demonstrates its potential for rapid phenotypic detection of bacteremia, thereby facilitating subsequent single‐cell AST to speed up turnaround time.

**FIGURE 5:**
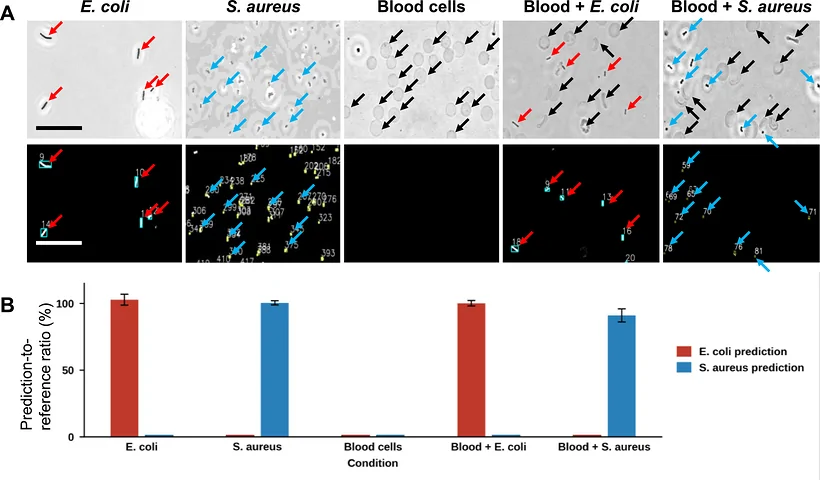
Assessment of U‐Net‐based bacterial detection across different biological conditions. (A) Representative phase‐contrast images of samples containing *E. coli* only, *S. aureus* only, blood cells only, *E. coli* mixed with blood cells, and *S. aureus* mixed with blood cells, together with the corresponding U‐Net–based prediction outputs. Individual cells are indicated by color‐coded arrows for visualization: red, *E. coli*; blue, *S. aureus*; black, blood cells. (B) Normalized bacterial identification and quantification across the five biological sample types. For each sample type, 10 independent images were analyzed, and U‐Net–based quantification was benchmarked against ground‐truth manual counts, with the prediction‐to‐reference ratio measured by the model‐predicted cell number divided by the corresponding ground‐truth manual count, expressed as a percentage. Scale bar: 10 µm. Data represent mean ± SD; *n* = 10.

## 3 Conclusion and Discussion

In this study, an integrated microfluidic‐AI platform for rapid pathogen diagnosis through single bacterial‐cell phenotypic analysis was developed. The system combines a simple, pressure‐balanced microfluidic device for stable confinement and time‐lapse monitoring of individual bacterial cells with a U‐Net–based image‐analysis pipeline for automated segmentation, counting, and interpretation of bacterial growth under antibiotic exposure. Using *Escherichia coli* as the primary model organism, we demonstrated rapid AST against CIP and SXT, with MICs determined within 2 h, which fully agreed with standard broth microdilution. In parallel, the AI module achieved nearly 100% training and validation performance, accurately quantifying 96% ± 1% of bacteria in independent test images with low false‐positive rates. The platform was further extended to complex biological conditions by successfully detecting 100% *E. coli* and 91% *S. aureus* cells in the presence of blood cells, with no non‐specific identification observed, supporting the feasibility of automated bacterial identification in biologically complex samples.

The key innovation of this work lies in the integration of two complementary pillars that together enable rapid pathogen diagnosis. The first pillar is single‐cell bacterial detection in a microfluidic environment capable of resolving bacterial growth and drug response within only a few cell cycles, thereby enabling the diagnostic window within a few hours rather than the days required for standard microbiology methods. The second pillar is AI‐empowered automation of single‐cell bacterial analysis that overcomes the labor‐intensive, non‐scalable, and error‐prone manual analysis bottleneck in current single‐cell diagnostic workflows for large image datasets, while yielding strong agreement with manual analysis for data tracking and AST interpretation. Moreover, the study suggests that this microfluidic‐AI automation can be adapted to conditions in which other infectious agents and complex biological components may be present, demonstrating its future feasibility.

Several important components remain to be investigated in future work. First, the single bacterial‐cell diagnostic capability should be expanded to a broader spectrum of clinically important pathogens, particularly ESKAPE organisms, and evaluated using antibiotics with distinct mechanisms of action to apply the platform across diverse bacterial physiologies, drug‐response phenotypes, and density‐dependent responses, including inoculum effects [54, 55]. Second, the AI framework should be further developed for species‐level classification and quantification beyond *E. coli* and *S. aureus*. Because these two organisms are morphologically distinct, the current results do not yet establish performance for species with closely overlapping appearances. Future studies should therefore include a broader range of bacteria, particularly morphologically similar species. Such expansion may be supported by the convolutional network's ability to learn latent image representations that may encode not only gross morphology but also edge structure, local texture and intensity patterns, spatial organization, and other image characteristics. Third, another limitation of the current study is that the blood‐matrix experiments were performed at bacterial concentrations higher than those typically found in direct bacteremia samples. Future integration with an upstream sample‐processing and concentration module for host‐cell depletion, bacterial concentration, and/or enrichment may therefore be necessary for applications to bacteremia diagnosis. Lastly, a higher level of systems integration will be needed to move from a microscopy‐based proof‐of‐concept to a practical diagnostic platform, including streamlined sample preparation, automated fluid handling, more unified image acquisition and analysis, and validation with larger numbers of clinically relevant samples. Together, these future studies will define how far this microfluidic‐AI strategy can advance from a promising prototype to a broadly applicable platform for rapid, automated pathogen diagnosis.

## 4 Materials and Methods

### 4.1 Microfluidic Device Fabrication

The microfluidic device was assembled from a glass substrate, double‐sided adhesive tape, a coverslip, and laser‐cut acrylic support layers (Figure 2b). All patterned layers were fabricated using a laser cutter (Epilog Laser Systems). The glass base provided optical transparency for microscopy and mechanical support for the multilayer structure. Microchannels were defined by laser‐cut double‐sided adhesive tape (Dwell GM4095T, Simin New Materials) with dimensions of 20 mm × 200 µm × 5 µm (L × W × H). Specifically, the 5 µm tape was first laminated between two layers of 3 M 468MP double‐sided adhesive tape, which served as sacrificial support layers to provide mechanical stability and prevent deformation or melting of the 5 µm tape during laser cutting. After cutting, these sacrificial layers, together with the protective liners of the 5 µm tape, were readily peeled off before the patterned middle tape layer was bonded to glass slides. The 200 µm channel width accommodates multiple bacteria within the same field of view while maintaining defined spatial confinement, and the 5 µm channel height—comparable to the diameter of a single bacterial cell (typically 1–2 µm)—enables those individual cells to remain in a narrow focal range and be individually resolvable by phase‐contrast microscopy. These dimensions are therefore specifically designed to support reliable single bacterial‐cell trapping, imaging, and quantification. After bonding to the glass substrate, the patterned tape was covered with a plastic coverslip to form enclosed microchannels while leaving the inlet and outlet open. A smaller acrylic frame was mounted on top of the coverslip to create a thin water layer above the designated channels, thereby improving thermal coupling and helping maintain the target incubation temperature during imaging. A larger acrylic fence separating multiple detection wells was then mounted on the device to create physically isolated testing regions. The assembled chip was heated at 70°C for 10 min to improve interlayer adhesion and obtain a leak‐resistant seal. Prior to use, the channel surfaces were rendered hydrophilic by air plasma treatment (PDC‐001, Harrick Plasma) for 5 min to improve sample loading and prevent bubble formation.

### 4.2 Bacterial Samples, Blood Samples, and Standard Microbiology Testing

*Escherichia coli* (ATCC 25922) and *Staphylococcus aureus* (ATCC 29213) were studied with standard microbiological procedures. Each bacterial species was inoculated into Mueller‐Hinton Broth (MHB), cultured at 37°C to reach the log phase, as indicated by an optical density at 600 nm (OD_600_) of approximately 0.2‐0.8, corresponding to a cell density of approximately 2—8 × 10^8^ CFU/mL.

Blood samples were obtained from BioIVT and adjusted to roughly 10^7^–10^8^ cells/mL in Mueller‐Hinton Broth. Human whole blood was kept at 4°C, and an aliquot was taken immediately before each experiment to ensure freshness. For bacterially spiked samples, all preparation steps were performed inside a biosafety cabinet to avoid contamination. The whole blood was first diluted 2‐fold in MHB, then a log‐phase bacterial suspension—vortexed briefly for uniformity—was added at a volume that achieved a final bacterial concentration of 10^7^–10^8^ CFU/mL in the blood‐MHB mixture. Each experiment used freshly prepared spiked samples. Before loading into the microfluidic device, the samples were gently mixed to prevent sedimentation. Imaging was performed using a phase‐contrast microscope (IX73, Olympus) equipped with a 40x objective and a CMOS camera (DP23M, Olympus) (Figure S1). Blood cells remained distinguishable from bacteria due to differences in size, shape, and contrast.

The standard microdilution method was used to determine resistance profiles and minimum inhibitory concentrations (MICs). Briefly, log‐phase bacteria were adjusted to 5 × 10^5^ cfu/mL and cultured at 37°C for 16–20 h in the presence of targeted antibiotic across two‐fold serial dilutions, following Clinical and Laboratory Standards Institute (CLSI) guidelines [56]. OD_600_ was monitored over time using a microplate reader (Synergy H1, BioTek) to evaluate bacterial growth and quantify the resistance profiles and MICs. Signal changes were normalized to the initial baseline.

### 4.3 Single Bacterial‐Cell Detection and AST

Freshly prepared log‐phase bacteria were diluted to 10^7^ colony‐forming units per milliliter (CFU/mL), and 2 µL of such samples was loaded into the microchannels by capillary action. Upon bacterial loading, additional medium was added to connect the inlet and outlet reservoirs, thereby suppressing convective flow and ensuring hydrodynamic balance within the channels. This pressure‐balanced condition minimized fluid motion in the channels, thereby facilitating the stabilization of individual bacterial cells inside. These individual bacteria were monitored using a motorized microscope (IX73, Olympus) equipped with a 40x objective and a CMOS camera (DP23M, Olympus), along with an in situ incubator set to 37°C (Tokai). Two to three spots were imaged from the middle of each channel, and a total of 6–9 images were captured from each device. For single bacterial‐cell antimicrobial susceptibility testing, *E. coli* samples were premixed with varying concentrations of designated antibiotics, including CIP and SXT, which were prepared to reveal resistance profiles and MICs following CLSI guidelines. Bacterial growth under antibiotic stress was monitored at the single‐cell level under the microscope for 2 h with a 30 min time‐lapse. Manual analysis of bacterial counts in targeted images was performed in ImageJ first, serving as the benchmark of single‐cell detection. Meanwhile, these images were processed for automated deep‐learning analysis, with no prior manual adjustment or interface.

### 4.4 U‐Net Architecture, Training, and Evaluation

A fully convolutional U‐Net architecture was adopted for automatic bacterial cell segmentation from phase‐contrast images. The network uses an encoder‐decoder structure with skip connections that preserve spatial information during multiscale feature extraction. In the contracting path, repeated 3 × 3 convolutions with ReLU activation are followed by 2 × 2 max pooling, progressively reducing spatial dimensions while increasing feature depth. Dropout regularization was applied during training, with dropout rates of 0.5, 0.4, 0.3, and 0.2 at successive network depths, to reduce overfitting and improve generalization across varying bacterial densities and imaging conditions. The bottleneck layer captures high‐level semantic information related to cell morphology, clustering, and overlap.

In the expanding path, transposed convolutions reconstruct full‐resolution segmentation maps. Feature maps from the encoder are concatenated with corresponding decoder maps through skip connections, allowing fine structural details to be recovered while preserving higher‐level contextual features. The final layer applies a 1 × 1 convolution with sigmoid activation to generate pixel‐wise probabilities for bacterial vs. background regions. The model was optimized with the Adam optimizer using binary cross‐entropy loss. The learning rate was adjusted through iterative tuning during model development: starting at 0.001, which led to loss oscillation and unstable convergence, it was then lowered to 0.000001, but the best‐optimized result was at 0.0001. All model training and inference took place on a Linux workstation with dual NVIDIA RTX A5500 GPUs (GA102GL architecture, 24 GB GDDR6 VRAM each). This setup delivered the necessary parallel computing power to efficiently train deep learning models over 500 epochs on a dataset of 7680 image patches.

The training dataset was prepared from in‐house‐generated microscopy images. The resolution of each microscopy image was 2076 × 3088 pixels, with each bacterial cell typically occupying only a small portion of the field of view (approximately 10 × 25 pixels). Thus, these full‐resolution images were divided into 128 × 128‐pixel patches to preserve local detail and improve learning efficiency. This patching strategy produced approximately 384 patches per image. The final training set included 19 images designed to cover a broad spectrum of conditions: 8 standard positive images showing typical bacterial densities, 5 augmented positive images created through manual cropping and zooming to introduce varied cell sizes and arrangements, 1 positive image with very few bacteria to enhance detection sensitivity at low cell counts, and 5 negative control images with no bacteria to minimize false positives. This selection ensured the model experienced the full range of conditions it would face in actual experiments. Together, these images yielded a dataset of 7296 128 × 128‐pixel patches. We trained the model using approximately 1600 to 1700 bacterial cells. Ground‐truth binary segmentation masks (bacteria “1” and background “0”) were manually generated in ImageJ [40] to distinguish bacterial cells from the background, which were then divided into matching patches and paired with the corresponding image patches for supervised training. In training, the dataset was divided into training (80%) and validation (20%) sets, and the model was trained for 500 epochs. Performance was evaluated using training accuracy, validation accuracy, training loss, validation loss, Dice coefficient, Jaccard index, recall, and precision. These metrics were used to assess both pixel‐level segmentation quality and the reliability of downstream bacterial detection.

### 4.5 Morphology‐Adaptive Quantification of Individual Bacteria

To quantify *Escherichia coli*, labeling of connected components (i.e., adjacent pixel values of 1) was applied to binary segmentation masks generated by the U‐Net model to identify individual bacterial regions. Each connected component was considered a candidate bacterium. The physical dimensions, such as the projected area of each bacterium, were determined through statistical measurements of over 30 manually annotated cells in ImageJ. Size thresholds were set at the mean ± 2× standard deviation, corresponding to a 95% confidence interval for single‐cell areas. These thresholds helped filter segmented objects, counting only regions consistent with single bacteria. Small artifacts below the threshold were removed as noise, while larger regions were excluded or examined further to avoid overcounting from overlapping cells.

To quantify *S. aureus*, which often appears as clustered cocci, a morphology‐adaptive algorithm was implemented. A similar thresholding method was applied to the binary images, followed by cleaning using connected‐component analysis and particle‐area filtering. For densely clustered regions, watershed segmentation was applied to improve the separation of neighboring cells. Object areas were then compared with the calibrated single‐cell size range to estimate bacterial counts. This combined strategy ensured accurate quantification of *S. aureus* without rebuilding the U‐Net model.

### 4.6 Automation of Single Bacterial‐Cell Detection and AST by the AI Model

The trained models were applied to independent test datasets to determine bacterial detection accuracy and counting performance. Automated bacterial identification and enumeration were benchmarked to manually derived ground‐truth analysis across all datasets. For AST, bacterial populations in control and antibiotic‐treated conditions were quantified over time to generate growth curves. Minimum inhibitory concentrations were defined as the lowest antibiotic concentrations at which no growth was observed during the 2‐hour observation window. These rapid, single‐cell–resolved measurements were then compared with manual analysis of the single‐cell assays and also standard broth microdilution results.

## Author Contributions

**Sabita Khadka**: methodology, software, investigation, Writing – original draft, Writing – review and editing. **Bushra Raahat**: methodology, investigation, writing – review and editing, software. **Spyros Tragoudas**: conceptualization, methodology, writing – review and editing, investigation. **Hui Li**: conceptualization, methodology, investigation, writing – review and editing, writing – original draft.

## Funding

Southern Illinois University (SIU) startup fund and the SIU System Collaborative Grant Award.

## Conflicts of Interest

The authors declare no conflicts of interest.

## Supporting information




**Supporting File**: advs77334‐sup‐0001‐SuppMat.pdf

## Data Availability

The data that support the findings of this study are available in the supplementary material of this article. The deep learning code and associated datasets are openly available on GitHub at https://github.com/Sabi2055/Bacteria‐counting.git
