# Supplementary material for: Artificial Intelligence‐Empowered Single‐Cell Phenotyping for Rapid, Automated Pathogen Diagnostics
Source: Adv Sci (Weinh). 2026 Aug 21;13(62):e77334. doi: 10.1002/advs.77334 (PMC13496276; doi:10.1002/advs.77334)
Supplement: Supplementary file 1 [file ADVS-13-e77334-s001.pdf]

Supporting Information

Artificial intelligence-empowered single-cell phenotyping for rapid, automated pathogen diagnostics

Sabita Khadka[1], *Bushra Raahat*[1], *Spyros Tragoudas*[1,*], *and Hui Li*[1,*]

[1]School of Electrical, Computer, and Biomedical Engineering, Southern Illinois University, Carbondale, IL, USA
E-mail: spyros@siu.edu and hui.li@siu.edu

Supplementary Materials

Figure S1. Experimental setup for single-cell bacterial detection using the microfluidic device.
Figure S2. Phase contrast time-lapse microscopy images of *E. coli* exposed to increasing ciprofloxacin (CIP) concentrations.
Figure S3. Phase contrast time-lapse microscopy images of *E. coli* exposed to increasing concentrations of sulfamethoxazole/trimethoprim (SXT).
Figure S4. Broth microdilution AST results for ciprofloxacin and trimethoprim/sulfamethoxazole against *E. coli*.
Figure S5. AI-predicted time-lapse microscopy images of bacteria exposed to increasing concentrations of ciprofloxacin (CIP).
Figure S6. AI-predicted time-lapse microscopy images of *E. coli* exposed to increasing concentrations of sulfamethoxazole/trimethoprim (SXT).
Figure S7. Validation of AI-based bacterial detection across sample matrices.
Figure S8. Assessment of AI-based bacterial detection across sample matrices.

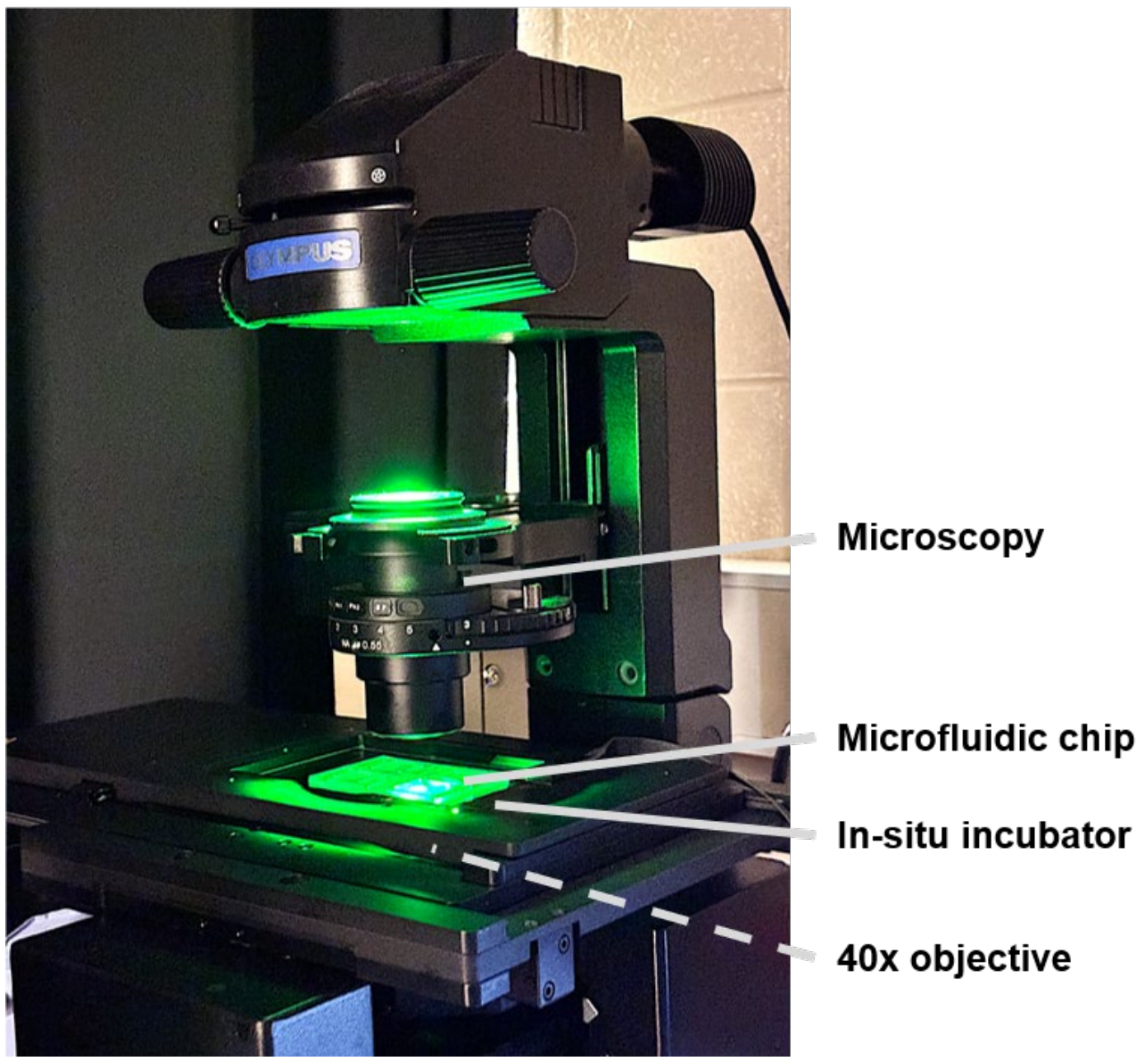

Figure S1. Experimental setup for single-cell bacterial detection using the microfluidic device. The microfluidic device was placed on a stage-top incubator mounted on an inverted microscope to maintain in situ imaging conditions during bacterial growth and antibiotic exposure. Images were acquired using a 40× objective and a CMOS camera.

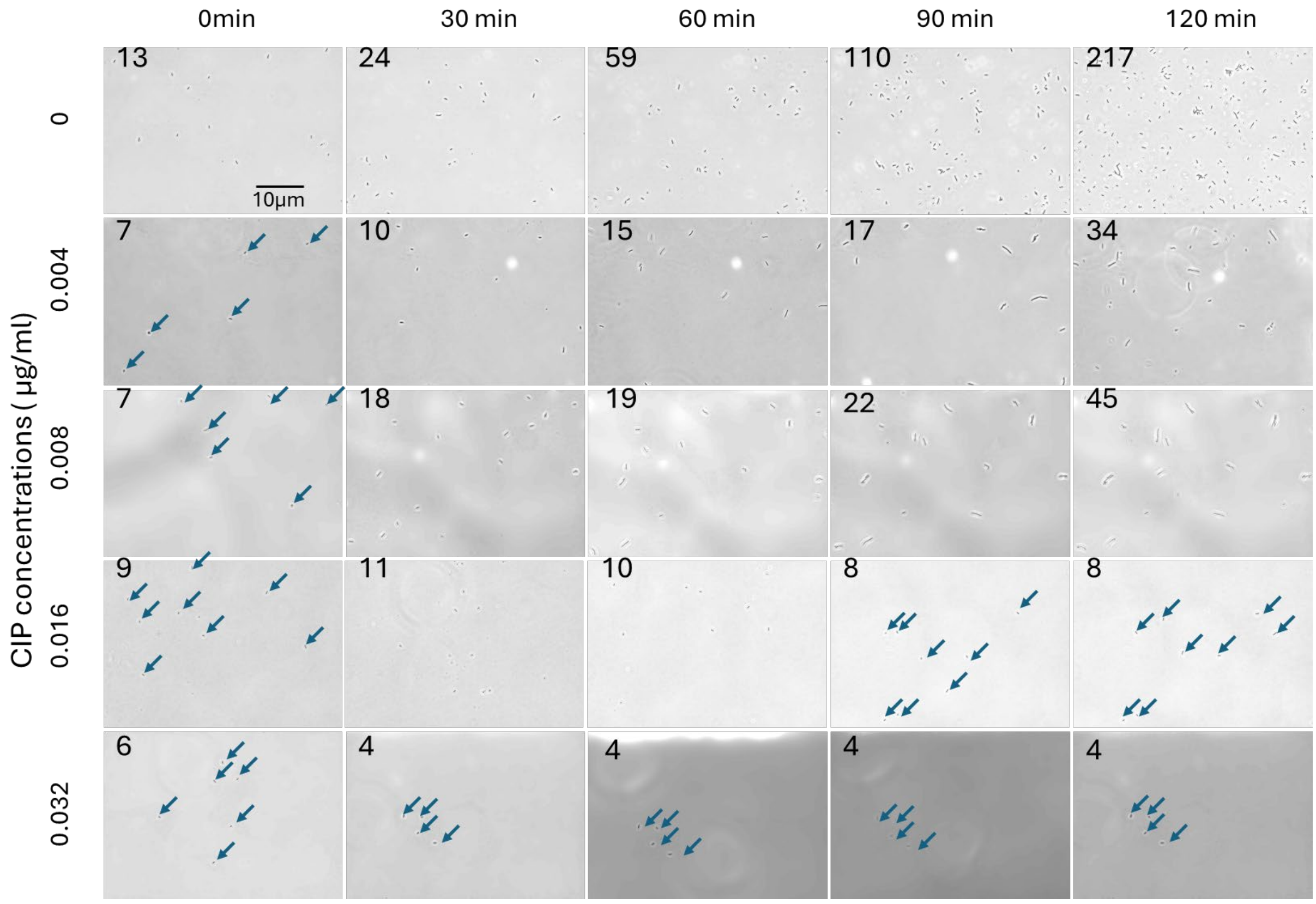

Figure S2. Phase contrast time-lapse microscopy images of *E. coli* exposed to increasing ciprofloxacin (CIP) concentrations. Each row shows a different CIP level (0, 0.004, 0.008, 0.016, and 0.032 µg/ml), while each column corresponds to a time point (0, 30, 60, 90, and 120 min). The top-left numbers in each image indicate the total *E. coli* cell count within that view. Blue arrows point to individual bacteria. Scale bar = 10 µm.

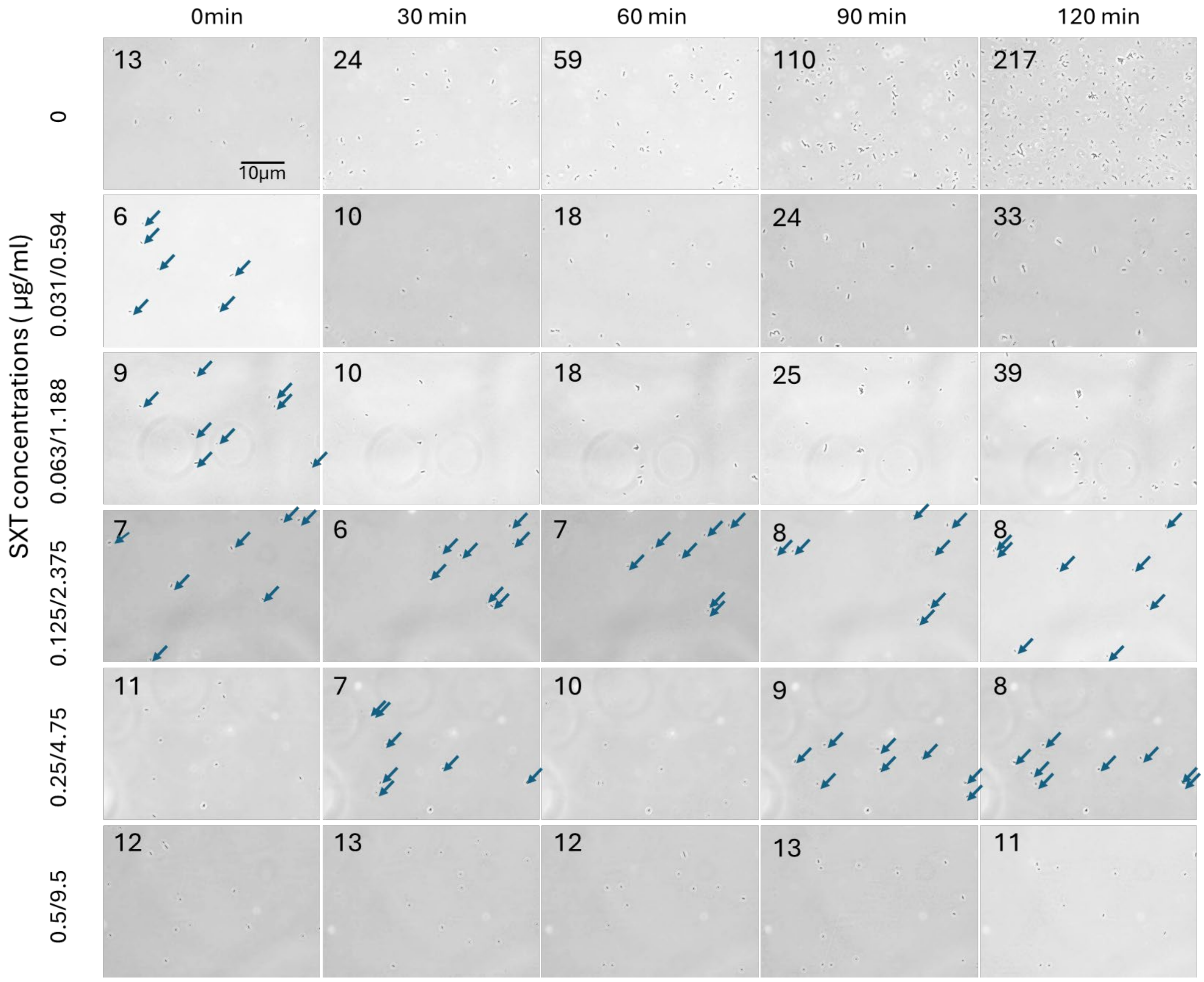

Figure S3. **Phase contrast time-lapse microscopy images of *E. coli* exposed to increasing concentrations of sulfamethoxazole/trimethoprim (SXT).** Each row corresponds to a specific SXT concentration (0, 0.03125/0.59375, 0.0625/1.1875, 0.125/2.375, 0.25/4.75, and 0.5/9.5 µg/ml), while each column depicts images at different time points (0, 30, 60, 90, and 120 minutes). The top-left numbers in each image indicate the total number of *E. coli* cells in the field of view. Blue arrows point to individual bacteria, and the scale bar represents 10 µm.

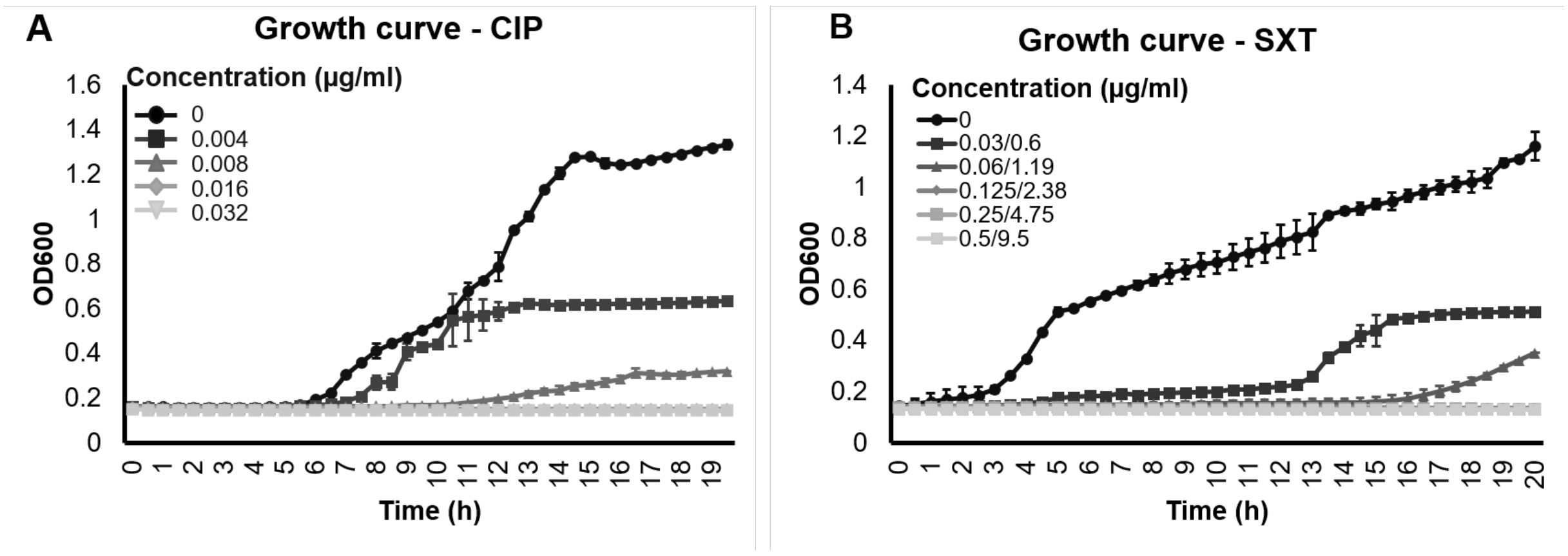

Figure S4. Broth microdilution AST results for ciprofloxacin and trimethoprim/sulfamethoxazole against *E. coli*. (**A**) Growth profiles of *E. coli* ATCC 25922 exposed to serial dilutions of ciprofloxacin (CIP), with the MIC determined as 0.016 µg/mL. (**B**) Growth profiles of *E. coli* ATCC 25922 exposed to serial dilutions of trimethoprim/sulfamethoxazole (SXT), with the MIC determined as 0.125/2.38 µg/mL. Data represent mean ± SD; *n = 2*.

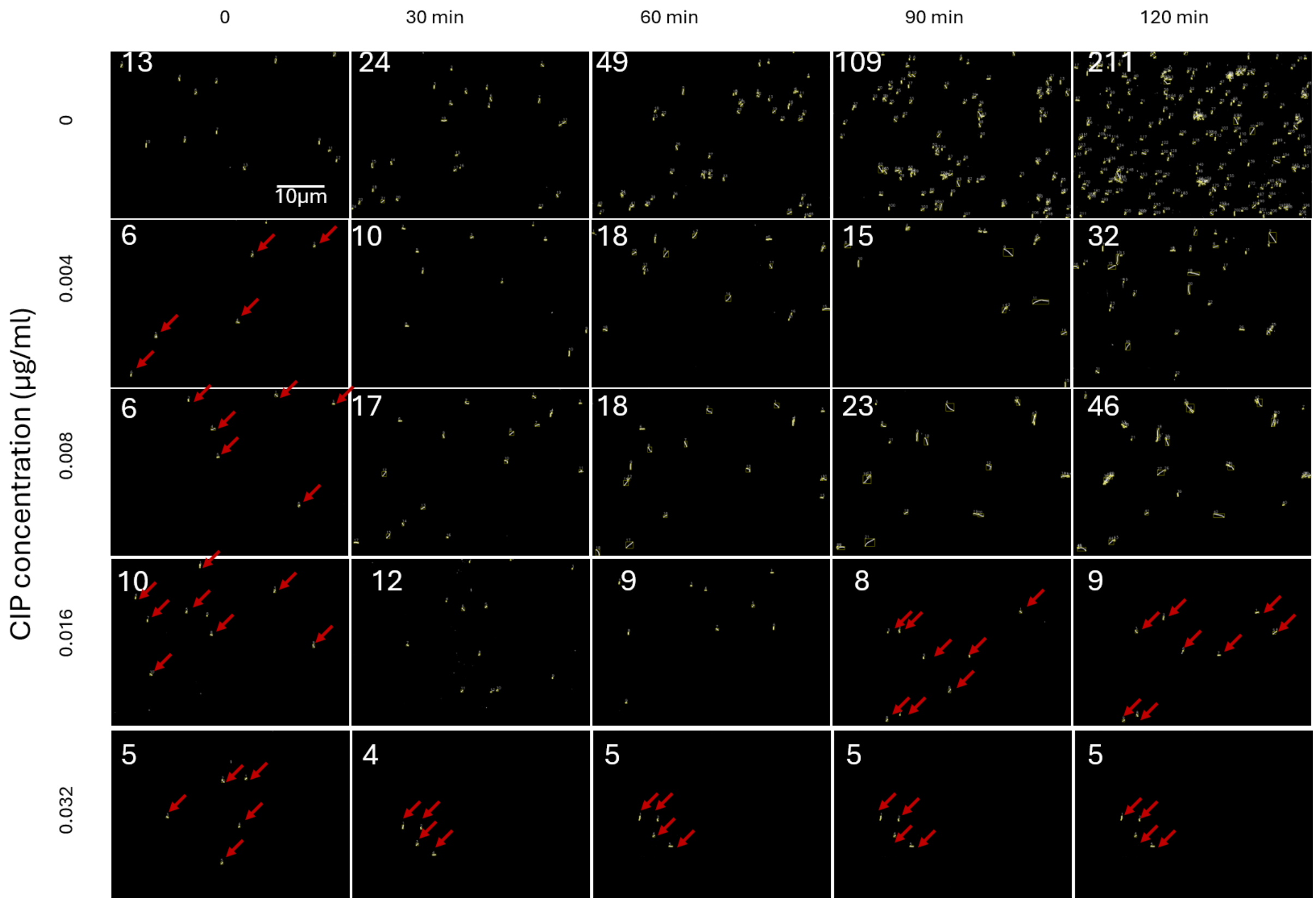

Figure S5. AI-predicted time-lapse microscopy images of bacteria exposed to increasing concentrations of ciprofloxacin (CIP). Each row corresponds to a specific CIP concentration (0, 0.004, 0.008, 0.016, and 0.032 µg/mL), and each column corresponds to a different time point (0, 30, 60, 90, and 120 minutes), corresponding to the images shown in Figure S1. The top-left corner of each image displays the total number of *E. coli* cells in that view. Red arrows point to individual bacteria, and the scale bar indicates 10 µm.

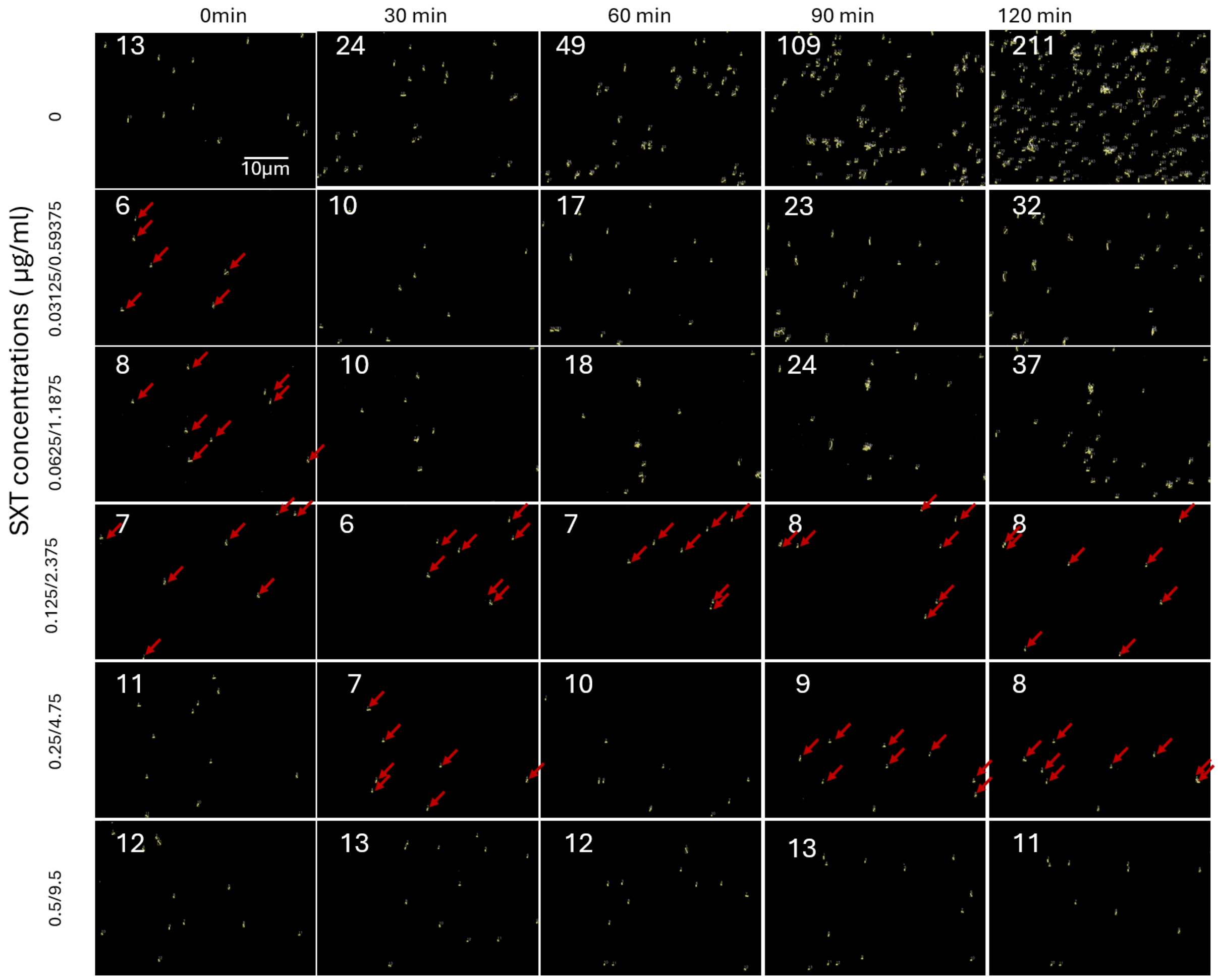

Figure S6. **AI-predicted time-lapse microscopy images of *E. Coli* exposed to increasing concentrations of sulfamethoxazole/trimethoprim (SXT).** Each row corresponds to a specific SXT concentration (0, 0.03125/0.59375, 0.0625/1.1875, 0.125/2.375, 0.25/4.75, and 0.5/9.5 μg/ml), while each column indicates a time point (0, 30, 60, 90, and 120 minutes), corresponding to the images shown in Figure S2. The top-left numbers in each image display the total *E. coli* cell count within the field of view. Red arrows point to individual bacteria, and a scale bar of 10 μm is included.

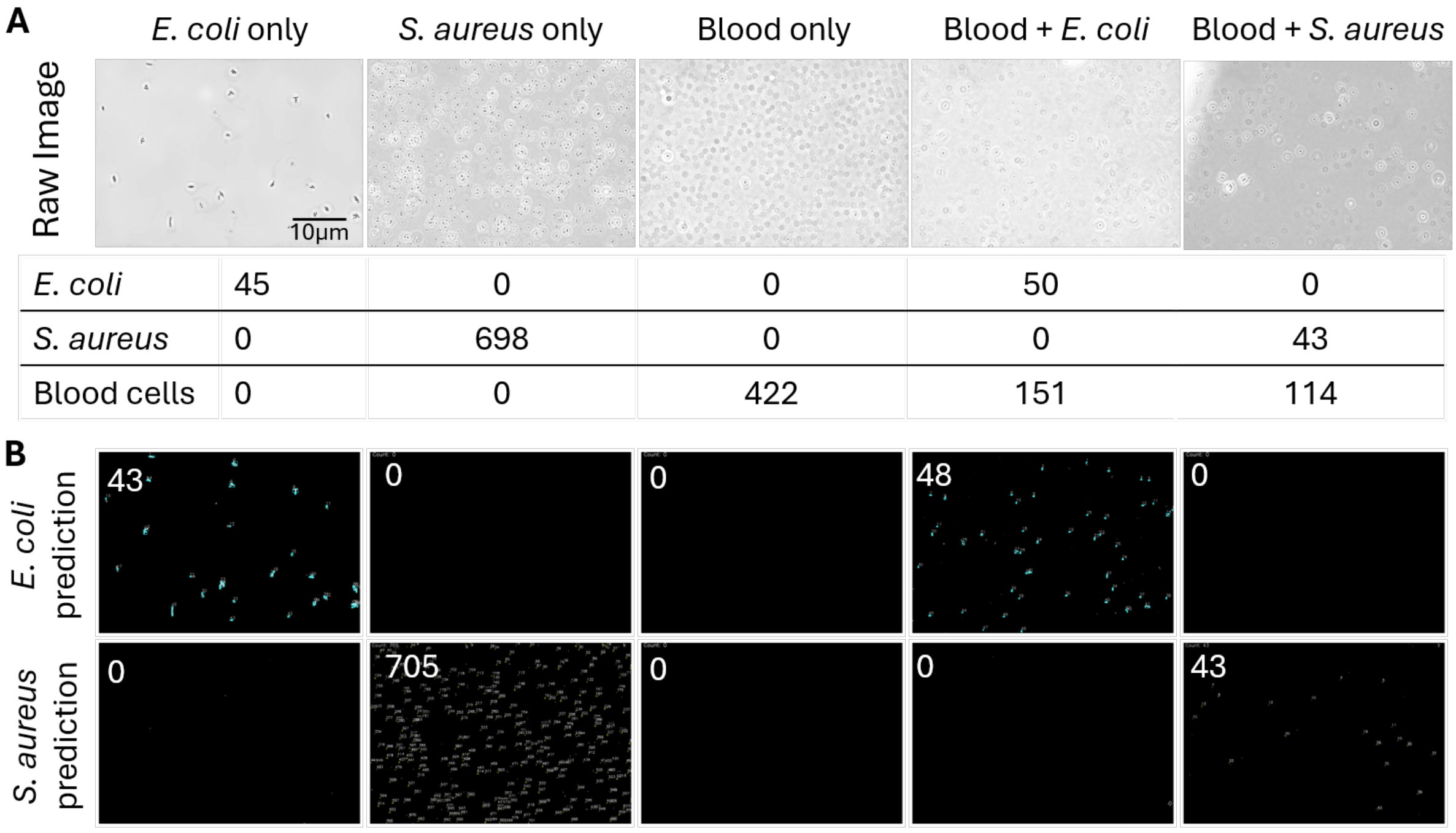

	E. coli only	S. aureus only	Blood only	Blood + E. coli	Blood + S. aureus
E. coli	45	0	0	50	0
S. aureus	0	698	0	0	43
Blood cells	0	0	422	151	114

Figure S7. Validation of AI-based bacterial detection across different sample matrices. (**A**) Representative microscopy images from five sample conditions: *E. coli* only, *S. aureus* only, blood cells only, blood cells mixed with *E. coli*, and blood cells mixed with *S. aureus*. Cell counts for each cell type and condition are provided in the corresponding table and served as ground-truth values for evaluating AI predictions. (**B**) Corresponding prediction masks generated by the *E. coli* model (top) and the *S. aureus* model (bottom). The predicted bacterial counts within each field of view are indicated by numbers. Scale bar: 10 µm.

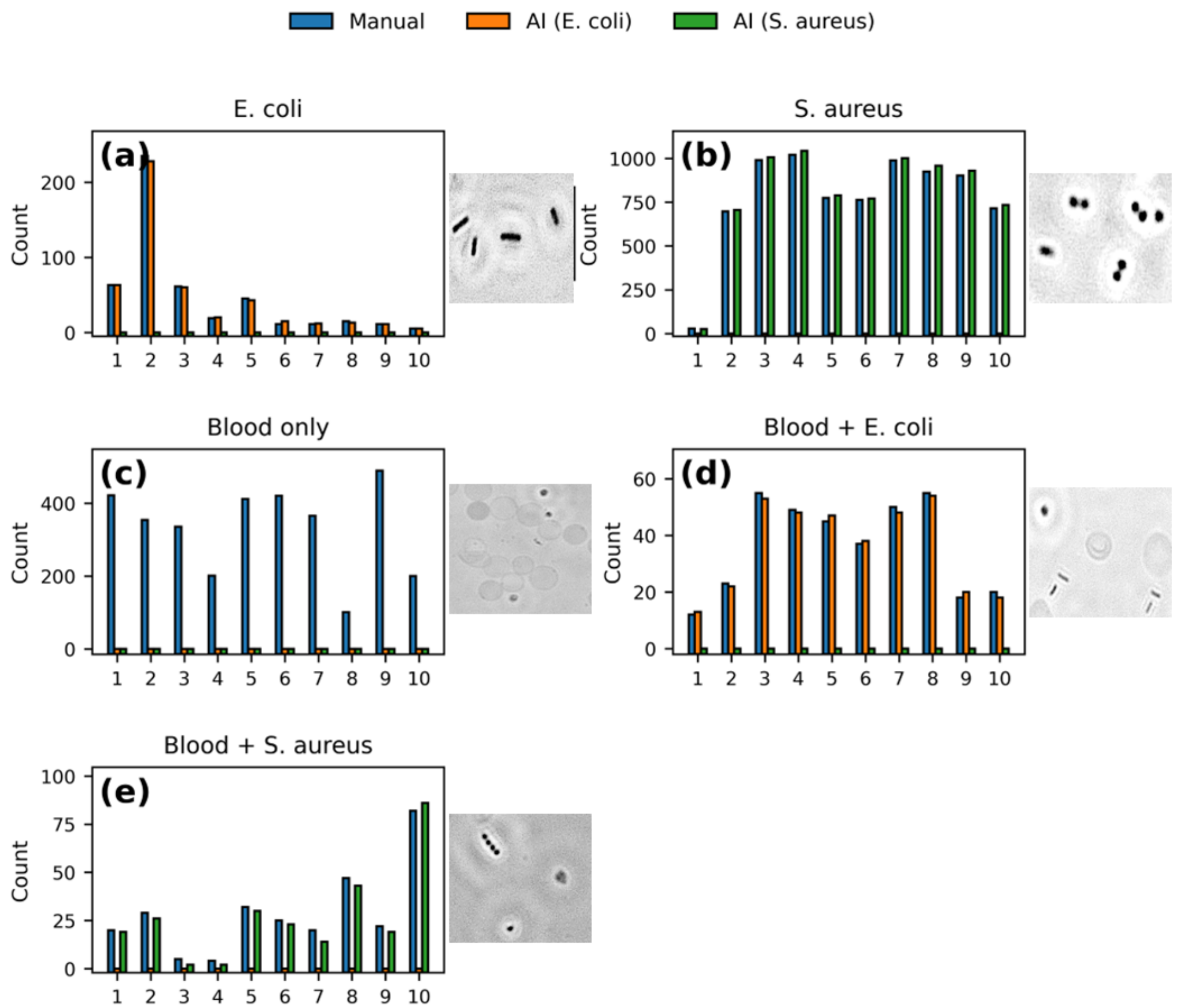

Figure S8. Assessment of AI-based bacterial detection across sample matrices. Bar charts compare manual cell counts with AI-predicted counts generated by the *E. coli* and *S. aureus* predictions across 10 fields of view for each sample condition without treatment with antibiotics: (**A**) *E. coli* only, (**B**) *S. aureus* only, (**C**) blood cells only, (**D**) blood cells mixed with *E. coli*, and (**E**) blood cells mixed with *S. aureus*. Manual counts, *E. coli* model predictions, and *S. aureus* model predictions are shown in blue, orange, and green, respectively. Representative images for each condition are shown alongside the corresponding quantification results.
